# Anuric Acute Kidney Injury Induced by Acute Mountain Sickness Prophylaxis With Acetazolamide

**DOI:** 10.1177/2324709614530559

**Published:** 2014-04-09

**Authors:** Javier A. Neyra, James Castle Alvarez-Maza, James E. Novak

**Affiliations:** 1 University of Texas Southwestern Medical Center, Dallas, TX, USA; 2 Clinica Anglo-Americana, Lima, Peru; 3 Henry Ford Hospital, Detroit, MI, USA

**Keywords:** acute kidney injury, acetazolamide, acute mountain sickness, anuria

## Abstract

Acetazolamide (ACZ) is a sulfonamide derivative that inhibits carbonic anhydrase and is the mainstay for prevention and treatment of acute mountain sickness (AMS). Acute kidney injury (AKI) is not well recognized as a complication of ACZ ingestion, especially when low doses are used for short periods of time. We report a case of a healthy, middle-aged man who developed severe AKI after the ingestion of ACZ for AMS prophylaxis. The patient presented with bilateral flank pain and anuric AKI without radiographic signs of obstructive uropathy. All blood and urine testing to determine the cause of AKI were negative or normal. The patient required 2 sessions of hemodialysis due to worsening metabolic derangements, which included severe anion gap metabolic acidosis and hyperphosphatemia. Renal function returned to baseline after 96 hours of supportive care. The pathogenesis of AKI in our patient was attributed to ACZ-induced sulfonamide crystalluria causing intratubular obstruction and retrograde urine flow, but not intraureteric precipitation or obstructive uropathy. This classic presentation of anuric AKI and renal colic has been previously described with higher doses of ACZ for prolonged periods of time but never with low doses for AMS prophylaxis such as in our patient (total dose of 1250 mg within 48 hours). Our case highlights the risk of adverse renal outcomes following ACZ ingestion, even in previously healthy individuals, and suggests that increased fluid intake may be advisable for travelers taking ACZ prophylaxis.

## Introduction

Ascent to high altitudes is common for both occupational and leisure activities. When the ascent is >2350 m, there is risk of high-altitude illness: acute mountain sickness (AMS), high-altitude cerebral edema, and high-altitude pulmonary edema. AMS is the most common form of high-altitude illness, and its incidence is about 50% at 2800 m.^1^ The main risk factor for high-altitude illness is an overly rapid ascent,^1^ so adherence to safe ascent rate recommendations is critical.^2^ Although individuals who typically ascend to high altitude are physically fit without major medical conditions, a careful evaluation to identify kidney, liver, and lung disease as well as to exclude pregnancy should be considered before ascent. Additionally, prophylactic and therapeutic agents must be dose-adjusted and interactions reviewed, particularly in the setting of other comorbidity or medications for traveler’s diarrhea or malaria.^3^

Acetazolamide (ACZ) is a sulfonamide derivative that inhibits carbonic anhydrase (CA) and was approved by the Food and Drug Administration in 1953 for its use as a diuretic, anticonvulsant, and antiglaucoma agent. ACZ is rapidly absorbed, achieves a peak concentration at 2 to 4 hours, and is eliminated unchanged in the urine. ACZ is also the mainstay for prevention and treatment of AMS.^4^ Evidence has shown that low doses of 250 mg per day may be equally effective compared to higher doses.^5^ We report a case of severe acute kidney injury (AKI), an underrecognized complication of ACZ, in a healthy middle-aged man after the ingestion of ACZ for AMS prophylaxis.

## Case Report

**Figure 1.**
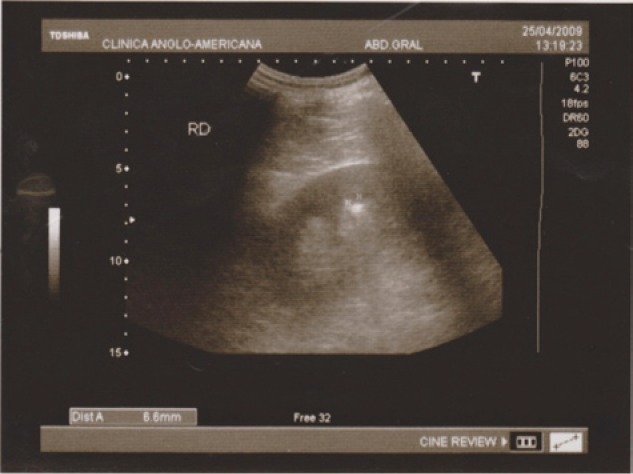
Renal sonography showing uncomplicated nephrolithiasis.

**Figure 2.**
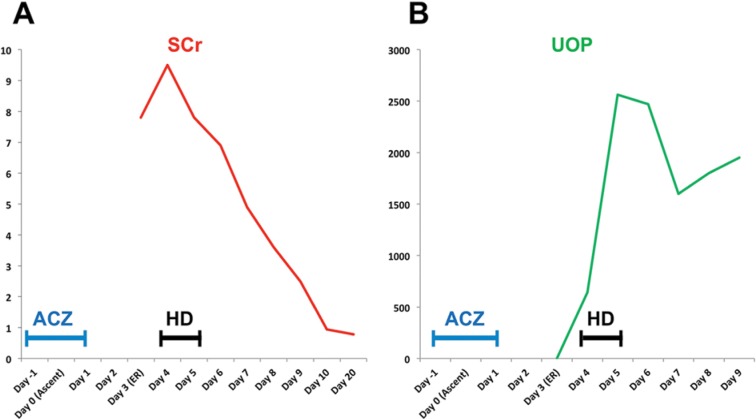
(A) Serum creatinine (SCr, mg/dL) trend in relation to acetazolamide (ACZ) exposure and hemodialysis (HD). (B) Urine output (UOP, mL) trend in relation to ACZ exposure and HD.

A 55-year-old businessman with a history of incidentally detected nephrolithiasis traveled to Peru. He took 2 doses of ACZ (Acetak) 250 mg before ascent to Ancash, Peru (4500 m above sea level) and 3 doses of 250 mg while at peak ascent, all at intervals of 12 hours (total dose of 1250 mg within 48 hours). He had taken similar prophylaxis without incident on previous trips to high altitudes. Twenty-four hours after peak ascent, he developed headache, nausea, bilateral low back pain, and oliguria. He received 1 injection of ketoprofen when he initially sought medical attention in an acute care center within the first hours of symptom onset. However, due to persistent symptoms, he returned to Lima, Peru (1550 m above sea level) to seek further medical attention. On presentation, his physical examination was unremarkable and vital signs were within normal limits. His initial evaluation included a kidney ultrasound that showed normally sized kidneys, uncomplicated right-sided nephrolithiasis without obstruction (3 small stones with diameters of 6.6 and 4 mm in the middle pole and 3.3 mm in the lower pole; Figure 1), and an empty bladder; the last was confirmed by bladder catheterization. Serum creatinine and blood urea nitrogen were 9.5 mg/dL and 94 mg/dL, respectively (Figure 2). Additional blood tests revealed mild leukocytosis, bicarbonate of 13 mmol/L, arterial pH of 7.27, arterial pCO_2_ of 30.5 mm Hg, anion gap of 31 mmol/L, potassium of 5.1 mmol/L, and phosphorus of 7.3 mg/dL. The patient received intravenous (IV) isotonic fluids (normal saline) with minimal response and subsequently required renal replacement therapy (2 sessions of hemodialysis) because of persistent anuria with worsening metabolic derangements (Figure 2). Additional workup included a negative renal Doppler ultrasound, negative serologies (rapid plasma reagin, hepatitis B surface antigen, hepatitis C antibody, human immunodeficiency virus-1 antibody, antinuclear antibody, anti-neutrophil cytoplasmic antibody, anti-streptolysin O antibody, and rheumatoid factor), normal serum complement levels, and no evidence of rhabdomyolysis (creatine phosphokinase and myoglobin within normal limits). Urinalysis showed urine pH of 6, specific gravity of 1010, fractional excretion of sodium of 22%, negative dipstick for albuminuria, and positive dipstick for blood. Urine microscopy revealed isomorphic red blood cells (RBCs; 12-15/hpf) and some leukocytes but no eosinophils, crystals, or granular casts. Renal function returned to baseline after 2 sessions of hemodialysis and 96 hours of supportive care and the patient was discharged in a stable condition. A 24-hour urine collection after AKI recovery revealed 0.24 g of protein and normal excretion of calcium, uric acid, sodium, phosphorus, and citrate. A kidney biopsy was not performed due to the rapid recovery of kidney function.

## Discussion

AMS occurs due to hypoxia-mediated mild cerebral edema and increased intracranial pressure^6^ or disruption of the blood–brain barrier causing vasogenic edema.^7^ Therefore, AMS symptoms are primarily caused by hypoxia and not hypobaria. ACZ increases minute ventilation by increasing tidal volume^8^ and improves arterial oxygenation and oxyhemoglobin saturation.^9^ The mechanism by which ACZ increases minute ventilation is not fully understood. It is possible that the metabolic acidosis caused by renal bicarbonate excretion attenuates the inhibitory effects of hypoxia-induced respiratory alkalosis.^10^ Also, bicarbonaturia creates a diffusion gradient between cerebrospinal fluid (CSF) and plasma bicarbonate concentration, ultimately lowering CSF pH and stimulating ventilation through central chemoreceptors.^11^ Additionally, inhibition of CA in red blood cells or vascular endothelial cells may cause carbon dioxide retention and tissue respiratory acidosis^12^ and thereby stimulate increased ventilation.^13^ Other therapeutic actions of ACZ include reduction in the number of bedtime arousals (apnea-associated hypoxemia) and improvement in the quality of sleep at high altitude by reducing periodic breathing (oscillations in respiratory frequency and/or tidal volume),^14,15^ an effect that is likely mediated by ACZ inhibition of peripheral chemoreceptors.^16^ Finally, the inhibition of renal CA induces water and salt losses in the kidney and may balance the state of increased aldosterone at high altitude, an adaptive response to plasma volume depletion and water losses from prolonged hyperventilation.^17^

The first description of renal lesions but not renal failure after treatment with ACZ was made by Glushien and Fisher^18^ in 1956 in a patient with Hodgkin’s disease who received a 4-day course (750 mg per day) of ACZ as a diuretic and was found to have coagulum and sulfonamide crystals in the renal tubules on postmortem analysis. Similarly, autopsy of a patient with nephrotic-range proteinuria who developed thrombocytopenic purpura and renal failure following exposure to ACZ as a diuretic (3000 mg in a period of 9 days) revealed dilated renal tubules containing casts and birefractive crystals, which resembled those obtained by recrystallizing ACZ in an acidic medium.^19^

In 1958, Yates-Bell^20^ described a case of “sulfonamide crystalluria” in a 54-year-old man exposed to ACZ for glaucoma (6-day treatment, dose not reported) who had severe low back pain, hematuria, azotemia, and anuria without evidence of kidney stones but with bilateral ureteral obstruction that required emergent catheterization. In this patient, the urinalysis revealed RBCs but not crystals or casts. The patient received oral bicarbonate therapy and recovered kidney function close to baseline by the time of discharge. In 1975, Howlett^21^ reported a case of a 69-year-old man who received a 2-week course of ACZ for glaucoma (1000 mg per day) and presented with anuria, azotemia, nausea, and flank pain. Urine microscopy showed many RBCs but not crystals on multiple evaluations. The patient received oral bicarbonate therapy and oral hydration and fully recovered kidney function. In 1978, 2 cases of “acute hemorrhagic anuria” following courses of ACZ (500 mg per day for 4 days and 1000 mg per day for 3 weeks to reduce intra-ocular pressure following cataract extraction and epilepsy, respectively) were reported. Both patients presented with back pain, progressed to anuria, and had antegrade urograms that revealed dilated renal pelvices and mucosal swelling or sludge-like material blocking the ureters. There was full recovery of kidney function following the release of the obstruction in both individuals.^22^

More recently, Epelde Gonzalo et al^23^ reported the case of a 76-year-old woman who was exposed to ACZ for glaucoma (425 mg per day for 3 weeks) and presented with anuric AKI without evidence of urologic obstruction or crystalluria. The patient responded to IV hydration and her kidney function returned to normal after 48 hours of supportive care. A similar case was described by Lopez-Menchero et al,^24^ in which a 58-year-old man who was taking ACZ for serous maculopathy (750 mg per day for 4 days) presented with low back pain and anuric AKI without radiographic evidence of obstruction or thrombosis. Urine microscopy showed RBCs but not casts or crystals. Again, the patient responded to IV hydration with normal saline and bicarbonate and fully recovered kidney function after 36 hours of hospital admission.

The most conclusive evidence of ACZ-induced AKI, confirmed by renal biopsy, was described in 1989 by Rossert et al^25^ in a 45-year-old man who was taking ACZ after a trabeculectomy of the left eye (500 mg per day for 2 days) and presented with anuric AKI, low back pain, and vomiting. There was no radiographic evidence of urologic obstruction on either renal ultrasound or intravenous pyelogram, and kidney biopsy revealed tubular damage with disrupted tubular basement membranes, cellular debris, and intratubular crystalluria. By immunofluorescence, heavy deposits of Tamm-Horsfall protein (THP) were detected in Bowman’s space of more than 50% of glomeruli. The patient responded well to supportive measures and significantly recovered kidney function by day 6 of hospital admission. THP is synthesized by the thick ascending limb of Henle’s loop and is typically present in the distal segments of the nephron. Therefore, its presence in the glomerulus may have been a marker of intratubular obstruction and retrograde flow of tubular urine caused by cellular debris and crystals.

Furthermore, there are several reports of calcium phosphate kidney stones apparently induced by the chronic use of ACZ.^21^ Although these patients often presented with renal colic and hematuria, AKI was not reported. For this reason, it is crucial to differentiate sulfonamide crystalluria after acute ACZ exposure as the cause of anuric AKI from calcium phosphate crystalluria most commonly attributed to chronic ACZ exposure. Therapeutic strategies should be adjusted to the specific clinical scenario. For example, sodium bicarbonate increases urine pH and would be expected to promote calcium phosphate precipitation and stone formation, but it may be useful to avoid precipitation of sulfonamide crystals (ACZ is insoluble in acidic urine, pK_a_ of 7.2).

In our patient, we hypothesize that the pathogenesis of AKI was related to ACZ-induced crystalluria causing intratubular obstruction and retrograde urine flow but not obvious intraureteric precipitation. Although ultrasonography is not sensitive enough to completely exclude intraureteric obstruction as a cause of anuric AKI, the clinical course (ie, no calculi passed and no radiographic evidence of bilateral nephrolithiasis) did not support obstructive uropathy secondary to nephrolithiasis as the primary cause of AKI.

Previous case reports have clearly documented that ACZ-induced anuric AKI may present with or without radiographic obstructive uropathy. In both clinical scenarios, the resolution of the obstruction^20,22^ and adequate, timely hydration^21,23,24^ are required for kidney recovery, which is often observed within the first 96 hours after ACZ discontinuation. The absence of a tissue diagnosis in most of the relevant literature corresponds to the rapid recovery of kidney function in most cases. Interestingly, urine microscopy has rarely demonstrated sulfonamide crystals in most of the cases of ACZ-induced AKI, including ours. The exposure to a single dose of ketoprofen was unlikely the cause of AKI in our patient, because acute interstitial nephritis is often non-oliguric and does not typically exhibit such a rapid renal recovery. However, it is possible that ketoprofen may have decreased renal blood flow and predisposed to sulfonamide crystal toxicity. Moreover, in the absence of major risk factors for nephrolithiasis, it is feasible that repeated exposure to ACZ may have predisposed our patient to the long-term formation of kidney stones as observed on ultrasound, although no objective evidence of calcium phosphate stones was obtained.

The classic presentation of anuria and renal colic (unilateral, bilateral, or alternating low back pain) has been described previously in patients receiving higher doses of ACZ for prolonged periods of time. However, ours is the first report of AKI after exposure to AMS prophylaxis with ACZ. Our case highlights the risk of adverse renal outcomes following ACZ ingestion even in previously healthy individuals and suggests that increased fluid intake and avoidance of nonsteroidal anti-inflammatory drugs may be advisable in travelers taking ACZ prophylaxis.
